# Comparison of Omadacycline and Tigecycline Pharmacokinetics in the Plasma, Epithelial Lining Fluid, and Alveolar Cells of Healthy Adult Subjects

**DOI:** 10.1128/AAC.01135-17

**Published:** 2017-08-24

**Authors:** Mark H. Gotfried, Karolyn Horn, Lynne Garrity-Ryan, Stephen Villano, Evan Tzanis, Surya Chitra, Amy Manley, S. Ken Tanaka, Keith A. Rodvold

**Affiliations:** a Pulmonary Associates, Phoenix, Arizona, USA; b College of Pharmacy, University of Illinois at Chicago, Chicago, Illinois, USA; c Paratek Pharmaceuticals, King of Prussia, Pennsylvania, USA; d College of Medicine, University of Illinois at Chicago, Chicago, Illinois, USA

**Keywords:** omadacycline, tigecycline, pharmacokinetics, epithelial lining fluid, alveolar macrophages, intrapulmonary penetration, lung

## Abstract

The steady-state concentrations of omadacycline and tigecycline in the plasma, epithelial lining fluid (ELF), and alveolar cells (AC) of 58 healthy adult subjects were obtained. Subjects were administered either omadacycline at 100 mg intravenously (i.v.) every 12 h for two doses followed by 100 mg i.v. every 24 h for three doses or tigecycline at an initial dose of 100 mg i.v. followed by 50 mg i.v. every 12 h for six doses. A bronchoscopy and bronchoalveolar lavage were performed once in each subject following the start of the fifth dose of omadacycline at 0.5, 1, 2, 4, 8, 12, or 24 h and after the start of the seventh dose of tigecycline at 2, 4, 6, or 12 h. The value of the area under the concentration-time curve (AUC) from time zero to 24 h postdosing (AUC_0–24_) (based on mean concentrations) in ELF and the ratio of the ELF to total plasma omadacycline concentration based on AUC_0–24_ values were 17.23 mg · h/liter and 1.47, respectively. The AUC_0–24_ value in AC was 302.46 mg · h/liter, and the ratio of the AC to total plasma omadacycline concentration was 25.8. In comparison, the values of the AUC from time zero to 12 h postdosing (AUC_0–12_) based on the mean concentrations of tigecycline in ELF and AC were 3.16 and 38.50 mg · h/liter, respectively. The ratio of the ELF and AC to total plasma concentrations of tigecycline based on AUC_0–12_ values were 1.71 and 20.8, respectively. The pharmacokinetic advantages of higher and sustained concentrations of omadacycline compared to those of tigecycline in plasma, ELF, and AC suggest that omadacycline is a promising antibacterial agent for the treatment of lower respiratory tract bacterial infections caused by susceptible pathogens.

## INTRODUCTION

Omadacycline (PTK 0796) is a semisynthetic derivative of minocycline and the first agent from the novel class of aminomethylcyclines. Similar to other tetracycline agents, omadacycline is a protein synthesis inhibitor which acts by binding to the 30S ribosomal subunit in the mRNA translation complex of bacteria and inhibiting the binding of aminoacyl-tRNA to the mRNA-ribosome complex (1–3). Omadacycline has shown potent *in vitro* antimicrobial activity against a wide spectrum of pathogens, including Gram-positive, Gram-negative, anaerobic, and atypical pathogens (1, 4–8). The chemical structure of omadacycline contains a unique alkylaminomethyl side chain at the C-9 position of the tetracycline D ring that enhances *in vitro* antibacterial activity against drug-resistant isolates, including strains expressing resistance through mechanisms of efflux and ribosomal protection (2, 9). This structure-activity relationship of omadacycline improves its *in vitro* antibacterial activity against resistant organisms, such as methicillin-resistant Staphylococcus aureus (MRSA) strains, penicillin- and multidrug-resistant Streptococcus pneumoniae strains, and vancomycin-resistant enterococci, over that of other tetracycline agents. Omadacycline also has antimicrobial activity against common extracellular respiratory pathogens, such as Haemophilus influenzae and Moraxella catarrhalis, and atypical pathogens, including Mycoplasma pneumoniae, Legionella pneumophila, and Chlamydophila pneumoniae (1, 4–8). Omadacycline is being developed for both intravenous (i.v.) and oral administration (1, 10–13). Phase 3 human clinical trials with omadacycline as monotherapy for the treatment of acute bacterial skin and skin structure infections (ClinicalTrials.gov registration no. NCT02378480 and NCT02877927) and community-acquired bacterial pneumonia (CABP) (ClinicalTrials.gov registration no. NCT02531438) have been conducted.

Epithelial lining fluid (ELF) and alveolar cells (AC) have been advocated as important infection sites for common extracellular and intracellular pathogens, respectively (14–24). Bronchoalveolar lavage (BAL) has become a standard method of determining extracellular and intracellular antibiotic concentrations after systemic administration of the antibiotic. Use of this method for the direct measurement of drug concentrations in ELF and AC, along with the concurrent determination of plasma drug concentrations, allows an approach to more knowledgeably evaluate the pharmacokinetics and exposure-response targets of antibiotics in lower respiratory tract infections.

The purpose of this study was to determine the concentrations of omadacycline and tigecycline in the plasma, ELF, and AC of healthy adult subjects. Tigecycline was used as a comparator because of its chemical structural similarity to omadacycline, its approved indication for the treatment of CABP, and its known intrapulmonary penetration. Comparison of the plasma and intrapulmonary pharmacokinetics of omadacycline and tigecycline was used to define the time course and magnitude of drug concentrations in the lung.

## RESULTS

### Subjects.

A total of 63 healthy adult subjects were enrolled and received at least one dose of either omadacycline (*n* = 42) or tigecycline (*n* = 21). Any subject that received at least one dose of either agent was included in the safety analysis population. One subject in the omadacycline treatment group whose BAL fluid was sampled at 2 h postdosing was not included in the pharmacokinetic analysis because of a BAL fluid sampling error. Two subjects receiving tigecycline discontinued the study treatment and the study due to adverse events. Two other subjects in the tigecycline treatment group did not have a bronchoscopy and BAL due to dosing errors. The remaining 58 subjects completed all study procedures and were included in the pharmacokinetic analysis population. The demographic characteristics of the subjects along with the total cell count in BAL fluid and the percentage of macrophages obtained from BAL fluid are listed in Table 1. The only notable differences between treatment groups were a slightly higher total cell count and percentage of macrophages in BAL fluid in the subjects receiving tigecycline.

**TABLE 1:** Characteristics of healthy adult subjects receiving omadacycline and tigecycline^*a*^

Treatment	Sex^*b*^ (no. of subjects)	Age (yr)	Ht (cm)	Wt (kg)	CL_CR_^*c*^ (ml/min)	Total cell count in BAL fluid (no. of cells/mm^3^)	% of macrophages in BAL fluid
Omadacycline (*n* = 41)	M (28), F (13)	38 ± 10	173 ± 10	78.0 ± 12.4	110 ± 21	128 ± 93	82 ± 17
Tigecycline (*n* = 17)	M (13), F (4)	40 ± 10	174 ± 10	78.6 ± 12.4	109 ± 26	154 ± 89	91 ± 5

a All data except for sex are expressed as the mean ± SD.

b M, male; F, female.

c CL_CR_, estimated creatinine clearance.

### Pharmacokinetics.

**FIG 1:**
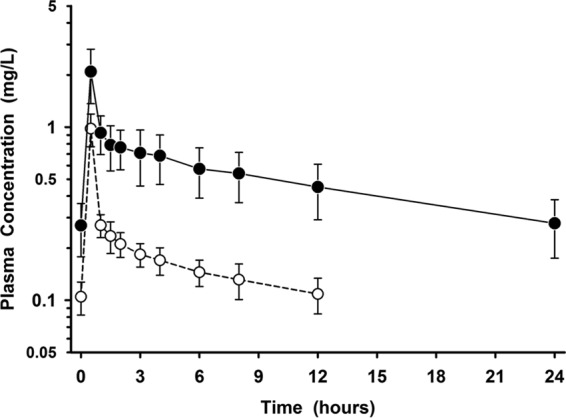
Mean ± SD plasma concentration-versus-time profiles before and after the final intravenous dose of omadacycline at 100 mg once daily (filled circles) and tigecycline at 50 mg every 12 h (open circles). The data on the *y* axis are on the log scale.

The mean ± standard deviation (SD) plasma concentration-versus-time profile after multiple intravenous doses of omadacycline at 100 mg and tigecycline at 50 mg are displayed in Fig. 1. One subject in the group whose BAL fluid was sampled at 1 h postdosing had a significantly higher measured omadacycline concentration in plasma prior to (time zero) the fifth intravenous dose of omadacycline than all other subjects (3.73 mg/liter versus a range of 0.125 to 0.59 mg/liter). This higher omadacycline concentration was not used for the assay whose results are presented in Fig. 1 or pharmacokinetic analysis. Instead, the plasma concentration obtained at the 24-h sampling time (0.237 mg/liter) was also used as a time zero value for this subject. The mean ± SD pharmacokinetic parameters of omadacycline and tigecycline from serial plasma concentrations are presented in Table 2. There were no significant differences in the values of the plasma pharmacokinetic parameters among the BAL fluid sampling groups for either omadacycline or tigecycline.

**TABLE 2:** Pharmacokinetic parameters for omadacycline (100 mg) and tigecycline (50 mg) in plasma infused i.v. over 30 min^*a*^

Treatment	*C*_max_ (μg/ml)	*C*_min_ (μg/ml)	AUC_0–tau_^*b*^ (μg · h/ml)	*V*_ss_ (liters)	CL (liters/h)	*t*_1/2_ (h)
Omadacycline^*c*^	2.12 ± 0.68	0.28 ± 0.10	12.14 ± 3.22	190 ± 53	8.79 ± 2.21	16.0 ± 3.5
Tigecycline^*d*^	0.98 ± 0.21	0.11 ± 0.03	2.20 ± 0.42	315 ± 67	23.1 ± 4.1	11.4 ± 2.6

a Data are expressed as the mean ± SD. Abbreviations: *C*_max_, maximum plasma concentration; *C*_min_, minimum plasma concentration; AUC_0–tau_, area under the plasma concentration-time curve for the dosing interval (tau); *V*_ss_, apparent volume of distribution at steady state; CL, apparent clearance; *t*_1/2_, elimination half-life.

b Tau (the end of the dosing interval) is 24 h for omadacycline (AUC_0–24_) and 12 h for tigecycline (AUC_0–12_).

c Parameter estimates for omadacycline in a total of 41 subjects following the fifth dose.

d Parameter estimates for tigecycline in a total of 17 subjects following the seventh dose.

**FIG 2:**
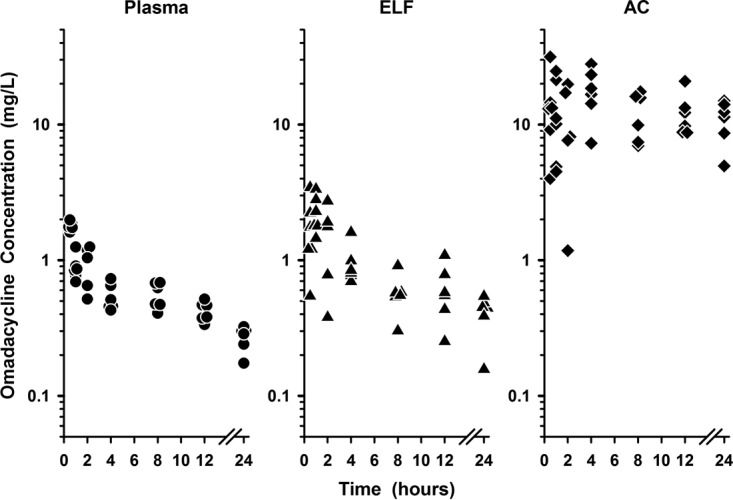
Individual concentrations of omadacycline in plasma (closed circles), epithelial lining fluid (ELF; closed triangles), and alveolar cells (AC; closed diamonds) at 0.5, 1, 2, 4, 8, 12, and 24 h after the fifth intravenous dose. The data on the *y* axis are on the log scale.

The individual plasma, ELF, and AC concentrations of omadacycline during the 24-h interval following the last dose are presented in Fig. 2. Once-daily intravenous dosing of omadacycline at 100 mg produced steady-state concentrations in ELF that were similar to or higher than the simultaneous plasma concentrations throughout the 24-h period after the last dose of omadacycline on the fifth day. In comparison, AC concentrations were significantly greater than the simultaneous plasma (or ELF) concentrations, and a majority of AC concentration values were maintained between 6 and 30 mg/liter throughout the 24-h period following the fifth dose of omadacycline. The mean values of the ratios of the ELF and AC concentrations to the simultaneous total plasma concentration for omadacycline during the 24-h period after drug administration ranged from 0.95 to 2.72 and 8.12 to 40.33, respectively.

**FIG 3:**
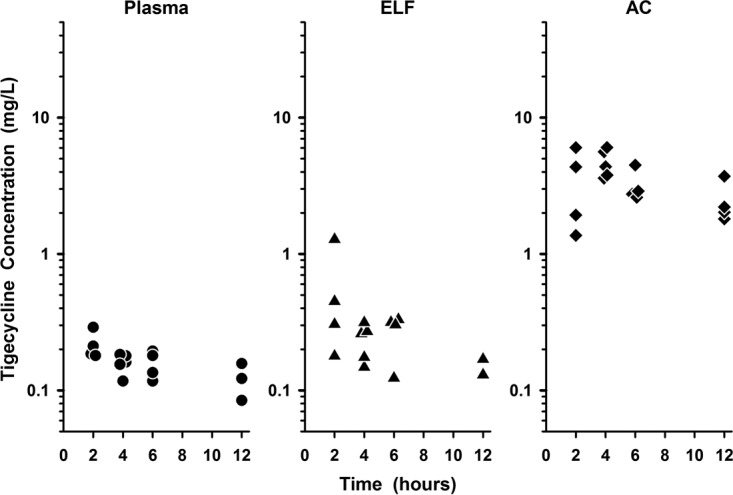
Individual concentrations of tigecycline in plasma (closed circles), epithelial lining fluid (ELF; closed triangles), and alveolar cells (AC; closed diamonds) at 2, 4, 6, and 12 h after the seventh intravenous dose. The data on the *y* axis are on the log scale.

**FIG 4:**
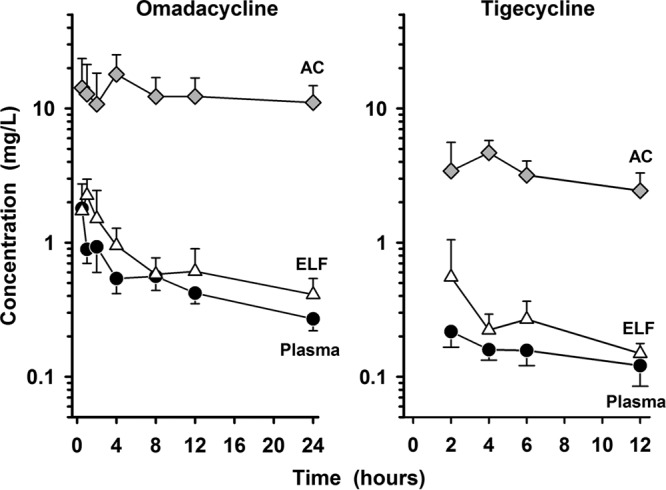
Mean ± SD plasma concentration-versus-time profiles of omadacycline (left) and tigecycline (right) in plasma (closed circles), epithelial lining fluid (ELF; open triangles), and alveolar cells (AC; shaded diamonds) after the last intravenous dose. The data on the *y* axis are on the log scale.

The individual plasma, ELF, and AC concentrations of tigecycline during the 12-h interval following the last dose are presented in Fig. 3. The magnitudes of the tigecycline concentrations in the three matrices were lower than those observed for the omadacycline concentrations (Fig. 2 to 4).

However, the pattern and time course of the tigecycline concentrations in all matrices were similar to those observed with omadacycline (Fig. 4). The intravenous administration of tigecycline at a loading dose of 100 mg followed by 50 mg every 12 h achieved ELF concentrations similar to or higher than the simultaneous plasma concentrations, and the AC concentrations were significantly higher than the plasma or ELF concentrations. The mean values of the ratios of the ELF and AC concentrations to the simultaneous total plasma concentration for tigecycline during the 12-h period after drug administration ranged from 1.47 to 2.30 and 14.89 to 29.94, respectively.

The mean ± SD concentrations of omadacycline after the fifth dose in plasma (total), ELF, and AC at the seven bronchopulmonary sampling times are reported in Table 3. The mean ± SD concentrations of omadacycline in plasma (total), ELF, and AC at the bronchopulmonary sampling times are illustrated in Fig. 4. The ELF and AC concentrations of omadacycline remained measurable at 24 h after the fifth dose with mean ± SD values of 0.41 ± 0.13 and 11.06 ± 3.72 mg/liter, respectively. The values of the area under the concentration-time curve (AUC) from time zero to 24 h postdosing (AUC_0–24_) based on mean and median ELF concentrations were 17.23 and 16.74 mg · h/liter, respectively. The ratio of the ELF to the total plasma omadacycline concentration based on the mean and median AUC_0–24_ values were 1.47 and 1.42, respectively. The AUC_0–24_ values based on the mean and median concentrations in AC were 302.46 and 292.31 mg · h/liter, respectively. The ratio of the AC to the total plasma omadacycline concentration based on the mean and median AUC_0–24_ values were 25.8 and 24.8, respectively.

**TABLE 3:** Omadacycline concentrations in plasma (total), ELF, and AC at time of bronchoscopy and BAL^*a*^

BAL fluid sampling time (h)^*b*^	Omadacycline concn (mg/liter)		
	Plasma	ELF	AC
0.5	1.80 ± 0.13	1.73 ± 1.01	14.26 ± 9.30
1	0.89 ± 0.19	2.25 ± 0.72	12.80 ± 8.48
2	0.93 ± 0.33	1.51 ± 0.94	10.77 ± 7.59
4	0.54 ± 0.12	0.95 ± 0.33	17.99 ± 7.17
8	0.56 ± 0.12	0.58 ± 0.19	12.27 ± 4.70
12	0.42 ± 0.07	0.61 ± 0.29	12.29 ± 4.61
24	0.27 ± 0.05	0.41 ± 0.13	11.06 ± 3.72

a Data are expressed as the mean ± SD at each sampling time. Abbreviations: BAL, bronchoalveolar lavage; ELF, epithelial lining fluid; AC, alveolar cells (macrophages).

b Samples were obtained from 6 subjects per sampling time except at the 2-h sampling time, when samples were obtained from 5 subjects.

The mean ± SD concentrations of tigecycline after the seventh dose in plasma (total), ELF, and AC at the four bronchopulmonary sampling times are shown in Table 4. The mean ± SD concentrations of tigecycline in plasma (total), ELF, and AC at the bronchopulmonary sampling times are displayed in Fig. 4. The mean ± SD tigecycline concentrations in ELF and AC remained measurable at 12 h after the seventh dose with values of 0.149 ± 0.028 and 2.44 ± 0.87 mg/liter, respectively. The values of the AUC from time zero to 12 h postdosing (AUC_0–12_) based on the mean and median ELF concentrations were 3.16 and 3.04 mg · h/liter, respectively. The ratio of the ELF to the total plasma tigecycline concentrations based on the mean and median AUC_0–12_ values were 1.71 and 1.66, respectively. The AUC_0–12_ values based on the mean and median concentrations in AC were 38.50 and 34.54 mg · h/liter, respectively. The ratio of the AC to the total plasma tigecycline concentrations based on the mean and median AUC_0–12_ values were 20.8 and 18.9, respectively.

**TABLE 4:** Tigecycline concentrations in plasma (total), ELF, and AC at time of bronchoscopy and BAL^*a*^

BAL fluid sampling time (h)^*b*^	Tigecycline concn (mg/liter)		
	Plasma	ELF	AC
2	0.217 ± 0.051	0.553 ± 0.497	3.42 ± 2.17
4	0.159 ± 0.026	0.233 ± 0.070	4.68 ± 1.10
6	0.157 ± 0.036	0.268 ± 0.097	3.18 ± 0.88
12	0.121 ± 0.036^*c*^	0.149 ± 0.028^*d*^	2.44 ± 0.87

a Data are expressed as the mean ± SD at each sampling time. Abbreviations: BAL, bronchoalveolar lavage; ELF, epithelial lining fluid; AC, alveolar cells (macrophages).

b Samples were obtained from 4 subjects per sampling time except at the 4-h sampling time, when samples were obtained from 5 subjects.

c Three of 4 subjects had plasma samples available for assay at this sampling time.

d Two of 4 subjects had concentrations equal to or above the quantitative level of detection.

### Safety and tolerability.

The overall incidence of treatment-emergent adverse events (TEAEs) and the most common TEAEs (those occurring in >1 subject in either treatment group) are shown in Table 5. All TEAEs were considered mild or moderate in severity. There were no serious adverse events reported in either treatment group during the study. TEAEs of epistaxis were not considered related to the study drug but to the bronchoscopy procedure, which was performed transnasally in most study subjects. The most notable difference between treatment groups was the incidence of nausea (2.4% in the omadacycline treatment group, 47.6% in the tigecycline treatment group). Two subjects were discontinued from tigecycline treatment and the study due to adverse events (both had nausea which was considered related to the study drug). No subjects receiving omadacycline were discontinued from either the study treatment or the study due to adverse events. There were no clinically significant differences between the treatment groups in analyses of laboratory values, electrocardiogram (ECG) parameters, or vital signs.

**TABLE 5:** TEAEs by preferred term that were reported in one or more subjects in either treatment group in the safety population

Adverse event	No. (%) of subjects with each adverse event who were given:	
	Omadacycline (*n* = 42)	Tigecycline (*n* = 21)
Subjects with at least one TEAE	12 (28.6)	11 (52.4)
Headache	5 (11.9)	3 (14.3)
Epistaxis	2 (4.8)	2 (9.5)
Nausea	1 (2.4)	10 (47.6)
Decreased appetite	0	2 (9.5)
Vomiting	0	3 (14.3)
Subjects with any TEAE leading to study drug discontinuation	0	2 (9.5)
Nausea	0	2 (9.5)
Subjects with any serious TEAE	0	0

## DISCUSSION

The pharmacokinetics of single and multiple intravenous doses of omadacycline in healthy adult subjects have recently been reported (10, 25). Following a single 100-mg intravenous dose, the mean ± SD plasma pharmacokinetic parameters of omadacycline were a maximum plasma concentration (*C*_max_) of 10.0 ± 1.5 mg/liter, an AUC from time zero to infinity (AUC_0–∞_) of 1.8 ± 0.7 mg · h/liter, and an elimination half-life (t_1/2_) of 16.8 ± 1.6 h (10). Following escalating single intravenous doses (25 to 600 mg), the values of *C*_max_ and AUC_0–∞_ increased in a dose-proportional and linear manner (25). In two cohorts of 10 healthy subjects receiving intravenous omadacycline at 200 mg once daily for 7 days, the mean values for *C*_max_ and AUC_0–∞_ ranged from 3.4 to 3.6 mg/liter and 28.9 to 30.9 mg · h/liter, respectively (25). The plasma concentrations and pharmacokinetic parameters observed in this study are similar to those observed after a single intravenous dose of 100 mg and multiple intravenous doses of 200 mg (after dose adjustment). The differences were relatively small and most likely explained by differences in gender (only one female was included in the other reported studies), dose administration schedules (200 mg once daily for seven doses versus 100 mg every 12 h for two doses followed by 100 mg once daily for three doses), and duration of therapy (7 versus 4 days).

A number of studies have evaluated the pharmacokinetics of intravenous tigecycline after the administration of single and multiple doses to healthy subjects and patients (26–30). The mean observed plasma concentrations and the values of the pharmacokinetic parameters for tigecycline in this study with healthy subjects were similar to those previously reported and outlined in the product package insert (*C*_max_, 0.98 versus 0.87 mg/liter; minimum plasma concentration [*C*_min_], 0.11 versus 0.13 mg/liter; AUC_0–24_, 4.40 versus 4.70 mg · h/liter; clearance [CL], 23.1 versus 23.8 liters/h) (31). Although the mean elimination *t*_1/2_ was only 11.4 h, whereas it is depicted to be 42.4 h in the product package insert, the lower value in this study most likely reflects the limited duration of blood sampling (i.e., 12 h) used during the final dosing interval.

This is the first study to investigate the intrapulmonary pharmacokinetics of omadacycline following multiple intravenous doses of 100 mg. The extensive BAL fluid sampling schedule for omadacycline was remarkable for illustrating intrapulmonary concentrations after the end of the final intravenous infusion and the accurate estimation of AUC values. The ELF concentrations during the first 1.5 h after the end of the infusion ranged from 0.36 to 3.44 mg/liter, with the majority (82%; 14/17) of these ELF concentrations being >1.2 mg/liter (Fig. 2). In addition, the ELF concentrations remained measureable for 24 h after the fifth dose (mean, 0.41 mg/liter; range, 0.16 to 0.54 mg/liter). The AUC_0–24_ ratios of the ELF to total plasma concentrations based on mean and median concentration values were 1.47 and 1.43, respectively. These values were slightly lower than those found if the ratios were based on the ELF and total plasma concentrations for each subject (mean, 1.59; range, 0.34 to 4.84). In comparison, the ratios of the AUC_0–12_ of the ELF to total plasma concentrations of tigecycline based on mean and median concentrations were 1.71 and 1.66, respectively. The ratios of the ELF and total plasma concentrations for each subject averaged 1.86 (range, 0.63 to 4.41). Although omadacycline and tigecycline have similar penetration ratios for ELF, the systemic exposure (e.g., the AUC_0–24_ values for both agents) to omadacycline in both ELF and plasma was approximately 3-fold higher than that to tigecycline. This is explained, in part, by the differences in plasma pharmacokinetic parameters, where CL and the apparent volume of distribution at steady state (*V*_ss_) for tigecycline were approximately 3 times and 50% higher, respectively, than those for omadacycline (Table 2). These differences remained true even when the extensive BAL fluid sampling for omadacycline was restrained to sampling times (e.g., 2, 4, 6 or 8, and 12 h) similar to those for tigecycline and if AUC_0–12_ values were compared or doubled in value (i.e., the AUC_0–24_ was used).

The ratio of the AUC_0–24_ to the MIC (AUC_0–24_/MIC) has been the pharmacokinetic-pharmacodynamic parameter that best correlates with antibacterial efficacy for the tetracycline class of antibiotics (30, 32–34). Lepak and colleagues have recently confirmed the relationship of the pharmacokinetic-pharmacodynamic parameters and the efficacy of omadacycline against four isolates of Streptococcus pneumoniae (range of MIC values, 0.0315 to 0.125 mg/liter) in a neutropenic mouse model of pneumonia (35). The ratio of AUC_0–24_/MIC for both unbound plasma and ELF had the highest correlation with efficacy (*r*^2^ ≃ 0.74). The typical range of ELF AUC_0–24_/MIC ratios associated with a net bacterial stasis and 1-log and 2-log reductions in the numbers of CFU from those at the baseline were 14.18 to 17.80, 6.00 to 17.61, and 17.26 to 47.27, respectively. The corresponding values for unbound plasma AUC_0–24_/MIC ratios were similar since the penetration of omadacycline from plasma into ELF approached 100% (range, 72% to 102%). Pfaller et al. have reported the *in vitro* activity of omadacycline against 6,253 isolates of Streptococcus pneumoniae (MIC_50_ and MIC_90_ = 0.06 and 0.06 mg/liter, respectively; MIC range, ≤0.015 to 0.5 mg/liter), including penicillin-intermediate strains (*n* = 1,040; MIC_50_ and MIC_90_ = 0.06 and 0.06 mg/liter, respectively) and penicillin-resistant strains (*n* = 1,466; MIC_50_ and MIC_90_ = 0.06 and 0.12 mg/liter, respectively) (5). *In vitro* activity of tigecycline against Streptococcus pneumoniae (MIC_50_ and MIC_90_ = 0.03 and 0.06 mg/liter, respectively) similar to that described above has been observed (1). In addition, omadacycline exhibited *in vitro* activity against tetracycline-sensitive and tetracycline-resistant strains of Streptococcus pneumoniae as well as *in vivo* efficacy against such strains in a mouse model of systemic infection (4). Combining these pharmacodynamic observations and MIC_90_ values with the observed mean ELF AUC_0–24_ value (17.23 mg · h/liter), the estimated AUC_0–24_/MIC ratio in ELF would be ∼287 and ∼144 for Streptococcus pneumoniae, respectively. Such high AUC_0–24_/MIC ratios of ELF in healthy subjects provide support for the regimen of intravenous dosing of omadacycline at 100 mg for the treatment of CABP caused by susceptible strains of Streptococcus pneumoniae.

The observed AC concentrations of both omadacycline and tigecycline were greater than the concurrent total plasma or ELF concentrations at all sampling times (Fig. 2 to 4). In addition, mean AC concentrations for both agents (Table 3 and 4) tended to remain constant throughout the dosing interval (Fig. 4). Similar penetration ratios of AC to total plasma concentrations were observed for omadacycline and tigecycline, no matter if the values were calculated with AUC ratios (with the AUC_0–24_ ratio, 25.8 and 24.8 on the basis of the mean and median concentration values, respectively for omadacycline; with the AUC_0–12_ ratio, 20.8 and 18.9 for tigecycline on the basis of the mean and median concentration values, respectively) or paired AC and total plasma concentrations for each subject (mean values, 23.2 for omadacycline and 22.2 for tigecycline). Omadacycline has potent *in vitro* activity against common atypical respiratory tract pathogens, including Legionella pneumophila (MIC_90_ = 0.25 mg/liter) and Chlamydophila pneumoniae (MIC_90_ = 0.25 mg/liter) (1, 7). Similar MIC_90_ values have been reported for tigecycline against Mycoplasma pneumoniae (MIC_90_ = 0.25 mg/liter) and Chlamydophila pneumoniae (MIC_90_ = 0.125 mg/liter), whereas MIC_90_ values for Legionella pneumophila ranged from 4 to 8 mg/liter (36). Nevertheless, tigecycline has been shown to be effective for the treatment of community-acquired pneumonia caused by Legionella pneumophila (37). Although a pharmacokinetic-pharmacodynamic parameter has not been established for intracellular concentrations of tetracyclines, the persistent and higher AC concentrations of omadacycline and tigecycline throughout the dosing interval are likely to contribute, in part, to the potency and clinical efficacy of these agents against intracellular pathogens in the lung.

Conte et al. also evaluated steady-state serum and intrapulmonary concentrations of tigecycline in 30 healthy adult subjects receiving the same intravenous dosage regimen (i.e., a loading dose of 100 mg followed by six 50-mg doses every 12 h) (28). Compared to the findings of this study, the range of mean concentrations of tigecycline in serum (0.10 to 0.19 mg/liter) and ELF (0.06 to 0.37 mg/liter) were more variable and slightly lower in value. The range of mean AC concentrations was significantly higher (10.7 to 15.2 mg/liter) and similar to the range of values observed for omadacycline. The differences in the ELF and AC concentrations of tigecycline between the two studies may be explained, in part, by differences in the analytical methods used to measure urea concentrations, the smoking status of the subjects, and the number of cells recovered in the BAL fluid. A subsequent population pharmacokinetic analysis with Monte Carlo simulations was performed with the serum and ELF concentrations observed by Conte et al. (28) plus 407 serum concentrations of tigecycline from four pharmacokinetic studies (38). Pharmacokinetic modeling of these data showed median values of AUC_0–24_ of 6.16 mg · h/liter for serum and 9.03 mg · h/liter for ELF. The median penetration ratio was 1.15 and displayed a wide range of variability (5th and 95th percentiles, 0.561 and 5.23, respectively).

Total (bound plus unbound) plasma concentrations of omadacycline and tigecycline were used to calculate penetration ratios in this study. Since the values reported for ELF and AC are assumed to represent unbound drug concentrations, total plasma concentrations are often corrected for protein binding and unbound plasma concentration are used to understand drug distribution and penetration. The mean plasma protein binding of omadacycline has been reported to be 20%, whereas the protein binding of tigecycline can range from 71% to 89% since tigecycline protein binding is concentration dependent (e.g., the unbound fraction is increased with an increase in the total concentration) (31, 39). For omadacycline, the respective ELF and AC penetration ratios based on unbound plasma concentrations would be 1.84 and 32.2, whereas those based on total plasma concentrations would be 1.47 and 25.8. Obviously, the reported penetration ratios would be greater if the unbound plasma concentrations were considered, particularly for highly protein-bound agents such as tigecycline. When total tigecycline concentrations in serum or plasma were used to calculate AUC values, the calculated ELF penetration ratios were 1.32 and 1.71 for the data reported by Conte et al. (28) and those presented in this study, respectively. These ratios dramatically increased to 6.59 and 8.54, respectively, after serum and plasma concentrations were adjusted for 80% protein binding. Such high values for the ELF penetration ratio (i.e., 6.17 and 8.07 at tigecycline doses of 25 and 50 mg/kg, respectively) have also been observed when unbound plasma concentrations were determined in an uninfected murine lung model (40).

The subjects enrolled in this study were healthy, nonsmoking male and female adults. Lipophilic agents similar to omadacycline and tigecycline usually demonstrate minimal to no changes in plasma concentration during clinical conditions of infection and inflammation since the apparent volume of distribution at steady state already has a large parameter value (i.e., 190 and 315 liters, respectively; Table 2). The observed concentrations in ELF and AC in this study serve as conservative estimates of the likely drug concentrations at extracellular and intracellular sites of the lung, respectively. Limited studies in animal models (40, 41) have suggested that the ratios of ELF to unbound serum concentrations were higher when tigecycline was administered to mice with pulmonary infections (12.9 and 23.3 in the two studies, respectively) than when it was administered to mice without pulmonary infections (6.2 and 8.1 in the two studies, respectively). In contrast, mean ELF concentrations of tigecycline were extremely low (range, 0.01 to 0.02 mg/liter) at 1, 4, and 12 h on the fifth day of a standard dosing regimen (i.e., a 100-mg loading dose followed by 50 mg every 12 h) in three critically ill patients treated for septic shock and ventilator-associated pneumonia (42). Regardless of the low ELF concentrations, mean AC concentrations and plasma pharmacokinetic parameters remained similar to those observed in our healthy subjects or by Conte et al. (28). Although differences in methodological procedures and physiological changes may partly explain the lower observed ELF concentrations, further studies are needed to appropriately translate and determine the reason for potential differences in the intrapulmonary concentrations of these agents in critically ill patients and healthy subjects.

In summary, this study provides pharmacokinetic and safety data for therapy with intravenous omadacycline (100 mg every 12 h for 2 doses followed by 100 mg every 24 h for 3 doses) and tigecycline (an initial 100-mg loading dose followed by 50 mg every 12 h for 6 doses) in healthy, nonsmoking male and female subjects. Omadacycline and tigecycline concentrations in plasma (total), ELF, and AC were observed to follow a similar pattern and a similar time course. The concentrations of both agents in ELF were similar to or slightly higher than the simultaneous total plasma concentrations throughout their respective dosing intervals. The concentrations in AC were significantly greater than those in both plasma and ELF, and the mean values of the AC concentrations for both agents remained relatively constant following administration of the last intravenous dose. Despite having similar penetration ratios on the basis of total plasma concentrations, the magnitude of the systemic exposure (based on AUC_0–24_ values) of omadacycline was approximately 3-fold higher than that of tigecycline in plasma (total), ELF, and AC. The frequency of adverse events was lower in subjects receiving omadacycline than in those receiving tigecycline. The results from this study lend support to exploring the use of omadacycline as a potential antibacterial agent for the treatment of CABP caused by susceptible strains of extracellular and intracellular pathogens.

## MATERIALS AND METHODS

### Study design.

This was a single-center, multiple-dose, open-label study in which healthy adult subjects were randomized to receive either omadacycline or tigecycline. Subjects were administered either omadacycline at 100 mg intravenously (i.v.) every 12 h for 2 doses and then every 24 h for 3 doses (5 total doses) or tigecycline at an initial loading dose of 100 mg i.v. followed by 50 mg i.v. every 12 h for 6 doses (7 doses total) in order to reach steady-state concentrations. These doses are the U.S. Food and Drug Administration (FDA)-approved dose of tigecycline for the treatment of CABP and the intended intravenous dose of omadacycline for the treatment of CABP; however, both drugs were administered for a shorter duration of therapy. Omadacycline was supplied by Paratek Pharmaceuticals, and tigecycline, which is manufactured by Pfizer (New York, NY), was purchased. The omadacycline in vials was reconstituted with 5 ml sterile water for injection and then added to a 100-ml normal saline (NS; 0.9% sodium chloride) infusion bag. The tigecycline in 50-mg vials was reconstituted with 5.3 ml NS and then added to a 100-ml NS infusion bag (the contents of 2 vials were added for the initial 100-mg dose). Both omadacycline and tigecycline were administered via a peripheral i.v. catheter, using a programmed infusion pump, over a duration of approximately 30 min. This study was approved by the Quorum Review Institutional Review Board, Seattle, Washington, USA. Written informed consent was obtained from all subjects prior to enrollment. Studies were conducted according to good clinical practice guidelines.

### Study population.

Subjects were eligible for enrollment if they were 18 to 55 years of age or older, weighed at least 50 kg, and had a body mass index (BMI) in the range of ≥18.0 and ≤30 kg/m^2^. An assessment of the subject's medical history, a physical examination, determination of vital signs, a 12-lead electrocardiogram (ECG), and laboratory tests were performed by a study physician prior to study entry. Any clinically significant abnormalities according to the opinion of the principal investigator or coinvestigator excluded the subject.

Subjects were excluded from participation if they met any of the following criteria: the subject was enrolled in any other investigational trials at the time of the baseline visit (or within 30 days or 5 half-lives of the study drugs, whichever was longer); the subject had a history of hypersensitivity to tetracycline antibiotics or a history of significant cardiac, neurological, thyroid, muscular, or immune disorders; the subject was currently pregnant or breast-feeding; the subject had used tobacco products in the last 3 months or had a positive urine cotinine test result; the subject had donated ≥400 ml of blood or plasma within 8 weeks prior to the baseline visit; the subject was HIV positive or had chronic hepatitis B or C virus infection; the subject had a history of drug or alcohol abuse within the past 12 months prior to dosing or evidence of drug abuse, as determined by laboratory assays of samples collected during the assessment; the subject had been treated with omadacycline or previously enrolled in this study; or the subject had abnormal laboratory test results at the baseline visit.

Female subjects had to have a negative serum pregnancy test result at screening and agreed to comply with the requirement to use a highly effective form of birth control from the screening visit through the final follow-up assessment. Male subjects agreed to use a highly effective method of birth control with a female partner(s) and to not donate sperm from the screening visit through the final follow-up assessment.

### Sample collection.

Blood samples were collected via a peripheral i.v. catheter placed in the arm contralateral to the site of infusion of the study medication. Blood samples were taken in order to obtain omadacycline concentrations in plasma immediately prior to the fifth dose (time zero) and at 0.5, 1, 1.5, 2, 3, 4, 6, 8, 12, and 24 h after the start of the fifth omadacycline infusion. Blood samples were collected in order to obtain tigecycline concentrations in plasma immediately prior to the seventh dose (time zero) and at 0.5, 1, 1.5, 2, 3, 4, 6, 8, and 12 h after the start of the seventh tigecycline infusion. Blood samples were collected into 4-ml tubes containing sodium heparin and then centrifuged for 10 min at approximately 4°C within 30 min of collection. The resulting plasma was divided into 2 equal aliquots, placed in cryovials, and frozen to −70°C within 1 h of collection until analytical analysis.

A single bronchoscopy and BAL were performed in each subject at either 0.5, 1, 2, 4, 8, 12, or 24 h following the start of the fifth dose of omadacycline and at either 2, 4, 6, or 12 h after the start of the seventh dose of tigecycline. Standardized bronchoscopy and BAL procedures have been previously described (14–24).

A blood sample was collected at the time of the 2nd BAL instillation in order to obtain a plasma urea concentration. Consecutive BAL fluid samples were collected and analyzed for urea concentration, cell count with differential, and either omadacycline or tigecycline concentrations. Sample collection and preparation techniques were performed in accordance with standardized procedures previously described (18–21).

### Analytical procedures.

Samples were prepared and omadacycline and tigecycline concentrations in plasma and BAL fluid were quantified using a validated method with liquid chromatography-tandem mass spectrometry (LC-MS/MS) analysis at Q^2^ Solutions (Ithaca, NY). The concentrations of omadacycline and tigecycline in cell pellets and the urea concentrations in plasma and BAL fluid were determined by LC-MS/MS at Keystone Bioanalytical, Inc. (North Wales, PA).

Deuterated forms of tigecycline (tigecycline-d_9_) and omadacycline (PTK 0796-d_6_) served as internal standards for determination of the concentrations of both drug analytes in plasma and BAL fluid, and [^13^C, ^15^N]urea served as the internal standard for determination of the urea concentration. Omadacycline was analyzed by reversed-phase solid-phase extraction for determination of the concentrations in both plasma and BAL fluid and by protein precipitation for determination of the concentrations in cell pellets. Tigecycline was analyzed by protein precipitation extraction for determination of the concentrations in plasma and cell pellets, and reversed-phase solid-phase extraction was used for determination of the concentrations in BAL fluid. Urea concentrations in plasma and BAL fluid were determined by extraction using protein precipitation and dilution, respectively.

### Plasma concentrations. (i) Omadacycline.

The lower limit of quantification (LLOQ) was 20 ng/ml, and the upper limit of quantification (ULOQ) was 2,000 ng/ml. The precision and accuracy for the quality control (QC) samples at concentrations of 60, 150, 758, 1,520, and 2,500 (diluted) ng/ml were ≤21.6% and 5.3 to 11.7%, respectively. The precision and accuracy for the calibration standard samples were ≤6.2% and −5 to 6.4%, respectively. Fifty-five of a total of 491 samples analyzed were selected for incurred sample reproducibility (ISR). The calibration curve for plasma assay validation was linear (*r*^2^ > 0.99). The samples were successfully analyzed within 7 runs. Precision and accuracy were met for the QC and calibration standard samples. ISR met the acceptance criteria.

### (ii) Tigecycline.

The LLOQ was 20 ng/ml, and the ULOQ was 2,000 ng/ml. The precision and accuracy for the QC samples at sample concentrations of 60, 600, and 1,500 ng/ml were ≤4.6% and 2.0 to 8.2%, respectively. The precision and accuracy for the calibration standard samples were ≤8.6% and −3.1 to 3%, respectively. Forty-five of a total of 188 samples analyzed were selected for ISR. The calibration curve for plasma assay validation was linear (*r*^2^ > 0.99). The samples were successfully analyzed within 3 runs. Precision and accuracy were met for the QC and calibration standard samples. ISR met the acceptance criteria.

### BAL fluid concentrations. (i) Omadacycline.

The LLOQ was 50 pg/ml, and the ULOQ was 2,000 pg/ml. The precision and accuracy for the QC samples at sample concentrations of 150, 600, 1,600, and 17,500 (diluted) pg/ml were ≤8.6% and −0.5% to 4%, respectively. The precision and accuracy for the calibration standard samples were ≤7.8% and −3.1% to 3%, respectively. ISR was determined to be not necessary for this study. The calibration curve for BAL fluid assay validation was linear (*r*^2^ ≥ 0.99). The samples were successfully analyzed within 3 runs. Precision and accuracy were met for the QC and calibration standard samples.

### (ii) Tigecycline.

The LLOQ was 1 ng/ml, and the ULOQ was 400 ng/ml. The precision and accuracy for the QC samples at sample concentrations of 3, 120, 300, and 1,000 (diluted) ng/ml were ≤3.7% and 7.7% to 10%, respectively. The accuracy of the calibration standard samples was −3.3% to 6.6% (precision was not done as the number of samples was ≤2). ISR was not performed, as there were insufficient samples for this evaluation. The calibration curve for BAL fluid assay validation was linear (*r*^2^ > 0.99). The samples were successfully analyzed within 1 run. Precision and accuracy were met for the QC and calibration standard samples.

### Cell pellet concentrations. (i) Omadacycline.

The LLOQ was 0.1 ng/ml, and the ULOQ was 100 ng/ml. The interassay precision and accuracy for the QC samples at concentrations of 0.3, 6, 75, and 1,500 (diluted) ng/ml were 1.78% to 9.85% and −3.67% to 2.03%, respectively. The intra-assay precision and accuracy for the QC samples were 1.71% to 8.97% and −5.83% to 4.07%, respectively. The interassay precision and accuracy for the calibration standard samples ranged from 0.48% to 4.32% and −4.25% to 4.75%, respectively. Twenty of a total of 42 cell pellet samples analyzed were selected for ISR. The calibration curve for cell pellet assay validation was linear (*r*^2^ = 0.99). The samples were successfully analyzed within 3 runs. Precision and accuracy were met for the QC and calibration standard samples. ISR met the acceptance criteria.

### (ii) Tigecycline.

The LLOQ was 0.1 ng/ml, and the ULOQ was 100 ng/ml. The interassay precision and accuracy for the QC samples at concentrations of 0.3, 6, 75, and 1,500 (diluted) ng/ml were 1.86% to 8.77% and −4.72% to 0.73%, respectively. The intra-assay precision and accuracy for the QC samples were 0.89% to 7.41% and −5.00% to 1.31%, respectively. The interassay precision and accuracy for the calibration standard samples ranged from 0.82% to 4.52% and −4.27% to 5.55%, respectively. Seventeen of a total of 17 cell pellet samples analyzed were selected for ISR. The calibration curve for cell pellet assay validation was linear (*r*^2^ = 0.99). The samples were successfully analyzed within 3 runs. Precision and accuracy were met for the QC and calibration standard samples. ISR met the acceptance criteria.

### Urea concentrations. (i) Plasma.

The LLOQ was 100 μg/ml, and the ULOQ was 3,000 μg/ml. The interassay precision and accuracy for the QC samples at concentrations of 155, 300, 1,000, and 2,250 μg/ml were 4.18% to 6.36% and −7.11% to −0.57%, respectively. The intra-assay precision and accuracy for the QC samples were 2.00% to 6.95% and −11.00% to −1.94%, respectively. The interassay precision and accuracy for validation of the plasma samples ranged from 1.24% to 3.80% and −6.07% to 2.98%, respectively. Twenty of a total of 59 plasma samples analyzed were selected for ISR. The calibration curve for the plasma assay validation was linear (*r*^2^ = 0.99). The samples were successfully analyzed within 2 runs. Precision and accuracy were met for the QC and calibration standard samples. ISR met the acceptance criteria.

### (ii) BAL fluid.

The LLOQ was 0.2 μg/ml, and the ULOQ was 10 μg/ml. The interassay precision and accuracy for the QC samples at concentrations of 0.6, 3, 7.5, and 30 μg/ml were 6.40% to 9.77% and −3.22% to 1.47%, respectively. The intra-assay precision and accuracy for the QC samples were 0.96% to 3.64% and −10.02% to 3.34%, respectively. The interassay precision and accuracy for validation of the plasma samples ranged from 1.03% to 6.61% and −3.03% to 5.38%, respectively. Twenty of a total of 59 BAL fluid samples analyzed were selected for ISR. The calibration curve for plasma assay validation was linear (*r*^2^ = 0.99). The samples were successfully analyzed within 3 runs. Precision and accuracy were met for the QC and calibration standard samples. ISR met the acceptance criteria.

### Calculation of ELF volume and antibiotic concentrations in ELF and AC.

The calculation of the ELF volume and the drug concentrations in ELF and AC was performed with BAL fluid supernatant and pulmonary (alveolar) cells (cell pellet) from aspirates recovered from the 2nd, 3rd, and 4th instillations (of a total of 4 instillations). The concentration of omadacycline or tigecycline in ELF (*C*_ELF_) was determined as follows: *C*_ELF_ = *C*_BAL_ × (*V*_BAL_/*V*_ELF_), where *C*_BAL_ is the concentration of omadacycline or tigecycline measured in BAL fluid, *V*_BAL_ is the volume of aspirated BAL fluid, and *V*_ELF_ is the volume of ELF sampled by BAL. *V*_ELF_ is derived from the following: *V*_ELF_ = *V*_BAL_ × (urea_BAL_/urea_plasma_), where urea_BAL_ is the concentration of urea in BAL fluid and urea_plasma_ is the concentration of urea in plasma (22).

The concentration of omadacycline or tigecycline in AC (*C*_AC_) was determined as follows: *C*_AC_ = *C*_pellet_ suspension/*V*_AC_, where *C*_pellet_ suspension is the amount of omadacycline or tigecycline measured in the 1-ml cell suspension, and *V*_AC_ is the volume of alveolar cells in the 1-ml cell suspension. *V*_AC_ was determined by multiplying the cell count in BAL fluid by the mean macrophage cell volume of 2.42 μl/10^6^ cells (23). The concentration of omadacycline or tigecycline measured in AC was derived from *C*_AC_ by adjusting for the percentage of macrophages in AC, as determined by a differential cell count of the BAL fluid (24). The concentrations in AC are the same as we have previously described for alveolar macrophages (18–21, 24).

The ratios of the ELF and AC concentrations to the simultaneous plasma concentrations were calculated for each subject and summarized for each group at each sampling time. The mean and median concentrations of omadacycline and tigecycline in BAL fluid obtained at different the bronchopulmonary sampling times (e.g., 0.5, 1, 2, 4, 8, 12, and 24 h for omadacycline and 2, 4, 6, and 12 h for tigecycline) were used to estimate the AUC_0–24_ for omadacycline and the AUC_0–12_ for tigecycline in plasma, ELF, and AC. The concentration at the final sampling time (24 h for omadacycline and 12 h for tigecycline) was also used as a value at time zero for determining the area term for plasma, ELF, and AC. The AUC values for each matrix were determined by the linear-log trapezoidal method. The ratios of the AUC of ELF to the AUC of plasma and the AUC of AC to the AUC of plasma were calculated.

### Pharmacokinetic analysis.

Noncompartmental analysis of omadacycline and tigecycline concentrations in plasma was performed using Phoenix WinNonlin (version 7.0) software (Pharsight Corp., Cary, NC). The maximum plasma concentration (*C*_max_) and the minimum plasma concentration (*C*_min_) were read from the observed plasma concentration-time profile following the fifth dose of omadacycline and the seventh dose of tigecycline. *C*_min_ was the plasma concentration 24 h after the fifth (last) dose of omadacycline and the plasma concentration 12 h after the seventh (last) dose of tigecycline. The AUC after the last dose was calculated by use of the linear-log trapezoidal method. The AUC for the last dose was determined for the 24-h dosing interval of omadacycline (AUC_0–24_ or AUC_0–tau_, where tau represents the end of the dosing interval) and the 12-h dosing interval of tigecycline (AUC_0–12_ or AUC_0–tau_). The elimination rate constant (β) was determined by nonlinear least-squares regression. The elimination half-life (*t*_1/2_) was calculated by dividing β into the natural logarithm of 2. The apparent clearance (CL) and apparent volume of distribution at steady state (*V*_ss_) were calculated with the following equations: CL = dose/AUC_0–tau_ and *V*_ss_ = MRTinf × CL, where MRTinf is the mean residence time extrapolated to infinity for infusion administration.

### Laboratory and safety assessment.

Subjects who received at least 1 dose of either omadacycline or tigecycline were included in the safety analysis. Safety assessments included physical examinations, ECG, vital sign determination, standard clinical laboratory evaluations (blood chemistry, hematology), pregnancy testing, and monitoring for adverse events (AEs) and serious adverse events (SAEs), which was performed during the course of the study. Safety data were summarized by treatment group, and the incidence of AEs was presented by system organ class and preferred term according to the *Medical Dictionary of Regulatory Activities* (MedDRA), the relationship to the study medication, and severity. Descriptive statistics of clinical laboratory, vital sign, and ECG results and the change from the baseline, as well as a summary of clinically notable values, were reviewed.
